# Local adaptation at higher trophic levels: contrasting hyperparasite–pathogen infection dynamics in the field and laboratory

**DOI:** 10.1111/mec.13928

**Published:** 2016-12-24

**Authors:** Steven R. Parratt, Benoit Barrès, Rachel M. Penczykowski, Anna‐Liisa Laine

**Affiliations:** ^1^ Metapopulation Research CentreDepartment of BiosciencesUniversity of HelsinkiViikinkaari 100014HelsinkiFinland; ^2^ Present address: Laboratoire de LyonUniversité de LyonANSES31 avenue Tony GarnierF‐69007LyonFrance; ^3^ Present address: Department of ZoologyUniversity of Wisconsin–MadisonMadisonWI53706USA

**Keywords:** co‐evolution, disease, host–parasite interactions, hyperparasite, local adaptation

## Abstract

Predicting and controlling infectious disease epidemics is a major challenge facing the management of agriculture, human and wildlife health. Co‐evolutionarily derived patterns of local adaptation among pathogen populations have the potential to generate variation in disease epidemiology; however, studies of local adaptation in disease systems have mostly focused on interactions between competing pathogens or pathogens and their hosts. In nature, parasites and pathogens are also subject to attack by hyperparasitic natural enemies that can severely impact upon their infection dynamics. However, few studies have investigated whether this interaction varies across combinations of pathogen–hyperparasite strains, and whether this influences hyperparasite incidence in natural pathogen populations. Here, we test whether the association between a hyperparasitic fungus, *Ampelomyces,* and a single powdery mildew host, *Podosphaera plantaginis,* varies among genotype combinations, and whether this drives hyperparasite incidence in nature. Laboratory inoculation studies reveal that genotype, genotype × genotype interactions and local adaptation affect hyperparasite infection. However, observations of a natural pathogen metapopulation reveal that spatial rather than genetic factors predict the risk of hyperparasite presence. Our results highlight how sensitive the outcome of biocontrol using hyperparasites is to selection of hyperparasite strains.

## Introduction

Parasites and pathogens pose a major risk to the stability of both natural and agricultural systems (Anderson & May 1991). Thus, determining the factors that influence the risk and severity of infection is vital to predict and control outbreaks of disease (Gilligan 2002; Shaw 2002; Zhan *et al*. 2014). Genetic diversity in both pathogen infectivity and host susceptibility traits can drive variation in disease outcome (Jackson & Tinsley 2005; Bangham *et al*. 2008), and infection success can vary among combinations of host–pathogen genotypes (Wolinska & King 2009). Selection can act on the variation among such G×G interactions to drive co‐evolutionary dynamics that further promote pathogen–host diversity and therefore influence the risks of infection. To quantify co‐evolutionary process in nature, many studies look for signals of local adaptation (LA), wherein fitness of either organism is higher when facing sympatric rather than allopatric antagonists (Blanquart *et al*. 2013). Local adaptation has been demonstrated for several host–pathogen systems (Thrall *et al*. 2002; Laine 2007; Schulte *et al*. 2011; Koskella 2014) and has been linked with disease dynamics and infection risk (Springer 2007; Tack *et al*. 2012; Kyle *et al*. 2014).

To date, almost all studies of G×G and LA in disease systems focus on the pathogen's interaction with its host or competing pathogen strains. However, pathogen dynamics can also be strongly influenced by hyperparasitism, wherein the pathogen itself becomes infected with a parasite. Hyperparasites are likely to be a common in nature, particularly among microorganisms (Parratt & Laine 2016), and theory has shown that they can fundamentally affect the ecological stability and evolutionary trajectory of the host–pathogen system in which they reside (Holt & Hochberg 1998; Taylor *et al*. 1998). Indeed, the limited studies of natural hyperparasites have shown that they can influence key epidemiological processes and thus greatly impact upon pathogen dynamics (Andersen *et al*. 2012; Springer *et al*. 2013; Tollenaere *et al*. 2014). However, despite being used as biological control measures in human medicine (Nobrega *et al*. 2015) and agriculture (Swinton & Gilligan 1999), the ecological, spatial and genetic factors governing hyperparasite infection success and epidemiology are poorly understood. In particular, whether or not the infection dynamic between hyperparasites and pathogens is governed by G×G interactions, and whether hyperparasites become locally adapted to their host is mostly unknown.

Hyperparasites are likely to be limited by the population size and distribution of the pathogens that they infect, which are in‐turn limited by the availability of their own host. This has the potential to select for a generalist lifestyle for many hyperparasites, or the ability to disperse much farther than their host in order to maintain an effective population size. Generalism is expected to prevent local adaptation and specialization between interacting species (Kawecki & Ebert 2004; Gómez *et al*. 2009); however, the impact of high dispersal levels on local adaptation is less clear. Multiple studies have shown that parasites with relatively higher gene flow than their host are most likely to become locally adapted (Greischar & Koskella 2007; Hoeksema & Forde 2008), but if gene flow becomes too strong then it can swamp local selection regimes, preventing local adaptation (Slatkin 1985; Lively 1999; Storfer 1999; Gandon & Michalakis 2002). Evidence for these effects is rare, but studies have found weaker genetic structure in hyperparasitoids compared to their parasite hosts, which alludes to a lack of divergence and specialization at the host‐deme level (Nair *et al*. 2016). However, environmentally dependent G×G interactions have been found in the interaction between a fungal pathogen and strains of its hyperparasitic virus (Bryner & Rigling 2011). If specific G×G interactions do occur between pathogens and hyperparasites in nature, then we may expect hyperparasites to become rare across fragmented pathogen populations and for their prevalence patterns to be aggregated around specific host genotypes. However, for most hyperparasite systems, simple descriptions of their prevalence, spatial aggregation and host associations are lacking particularly among pathogen individuals or demes of the same species.

Here, we investigate the influence of genetic and ecological factors on the prevalence and infection dynamics of the hyperparasitic fungus *Ampelomyces spp*, an antagonist of powdery mildew pathogens. *Ampelomyces* is considered to be a group of generalist hyperparasites (Kiss & Nakasone 1998) as several cross‐inoculation studies have found that isolates are able to infect multiple mildew species (Szentivanyi *et al*. 2005; Liang *et al*. 2007; Kiss *et al*. 2011), and single mildews can become infected with phylogenetically distinct *Ampelomyces* strains (Pintye *et al*. 2012). However, molecular evidence suggests that *Ampelomyces* lineages are broadly associated with specific mildew host species in nature (Liang *et al*. 2007; Park *et al*. 2010; Kiss *et al*. 2011). Microsatellite analysis has demonstrated that this divergence may be driven by host phenology in some instances (Kiss *et al*. 2011; Pintye *et al*. 2015). In addition, *Ampelomyces* isolated from different host species have been shown to vary in their growth rate, morphology, spore germination response *in vitro* and in their infection severity against different powdery mildew species (Legler *et al*. 2011, 2016; Angeli *et al*. 2012a). This evidence suggests that some degree of host association and co‐evolution may occur within *Ampelomyces,* although strict host‐species fidelity has not evolved. To date, there is little demonstration of any G×G interactions or local adaptation between *Ampelomyces* and genetically distinct isolates of a single mildew species. The existence of such dynamics could have profound impact for both the hyperparasite's epidemiology and the disease dynamics of the mildew itself.

In this study, we test whether *Ampelomyces* exhibits G×G infection dynamics and local adaptation across a metapopulation of a single host species: *Podosphaera plantaginis*. We use cross‐inoculation laboratory studies to expose variation in infection dynamics among hyperparasite and pathogen combinations, and to contrast infection success between sympatric and allopatric pairings. We also test whether hyperparasite infection in the field is clustered by pathogen genotypes, as would be expected if G×G effects are prevalent, or if ecological properties of the mildew host best describe patterns of *Ampelomyces*. Finally, we contrast hyperparasite prevalence at the pathogen population and metapopulation scales. Our results demonstrate that pathogen and hyperparasite genotypes, as well as their interaction, determine the outcome of infection in the laboratory. We also observed signals of local adaptation that vary in strength among hyperparasite strains. However, these genotypic effects were not readily apparent in the field, primarily due to a strong spatial aggregation of the hyperparasite at the local level.

## Methods

### Study system

*Podosphaera plantaginis* (Castagne; U. Braun & Takamatsu) is a specific and obligate fungal pathogen of ribwort plantain (*Plantago lanceolata*) that causes powdery mildew disease on above ground tissues of the host*. Po. plantaginis* is aerially dispersed as haploid, asexually produced spores (conidia) during the spring and summer. *Po. plantaginis* typically undergoes between six to eight clonal cycles, which lead to local epidemics of powdery mildew symptoms. The mildew overwinters as sexually produced [through outcrossing or selfing (Tollenaere & Laine 2013)] resting structures called chasmothecia [formerly cleistothecia (Braun 1995)] that are produced towards the end of the growing season (August–September). *Pl. lanceolata* is a perennial herb, capable of reproducing clonally through vegetative growth and sexually via wind and animal dispersed seeds. In the Åland archipelago (SW Finland), *Pl. lanceolata's* preferred dry meadow habitat is fragmented into well‐defined, discrete populations (Ojanen *et al*. 2013), which support a classical metapopulation of the powdery mildew pathogen (Laine 2004; Laine & Hanski 2006). Annual surveys of the metapopulation since 2001 have revealed that a balance of local extinction and colonization events maintain pathogen prevalence at 1.1–16.9% of the *c*. 4000 host populations among years (Jousimo *et al*. 2014).

*Ampelomyces* spp are obligate mycoparasites of multiple powdery mildew species belonging to the *Erysiphaceae*, including *Po. plantaginis* (Kiss *et al*. 2004). *Ampelomyces* hyphae invade the tissues and cells of established powdery mildew lesions where they absorb nutrients and degenerate the host cytoplasm. *Ampelomyces* hijacks the mildew's reproductive structures in which it established pycnidia; sacks of hyperparasite spores that are then released and dispersed aerially and through rain splash to neighbouring mildew hosts (Sullivan & White 2000). The infection process (spore germination–pycnidia formation) takes between 5 and 8 days under laboratory conditions; thus, *Ampelomcyes* is likely to have a shorter clonal generation time than its host in nature. However, the true within‐host rate of reproduction and the existence or timing of any sexual recombination remains unknown (Kiss *et al*. 2004).

In the Åland *Po. plantaginis* metapopulation, *Ampelomyces* infection has been shown to significantly reduce the probability that the mildew will successfully overwinter (Tollenaere *et al*. 2014). This is most likely due to the hyperparasite's ability to suppress the formation of powdery mildew chasmothecia (Caffi *et al*. 2013). Furthermore, in other mildew hosts, *Ampelomyces* has been shown to have negative effects on within‐season mildew growth, reducing the number and viability of the mildew's asexual conidia (Falk *et al*. 1995; Abo‐Foul *et al*. 1996; Verhaar *et al*. 1996; Shishkoff & McGrath 2002; Romero *et al*. 2003). This can lead to the collapse of powdery mildew lesions and curtail its spread among plant populations. Given that within‐season transmission is likely to be a major component of mildew fitness [infection level at the end of the epidemic is also an important determinant of mildew overwintering success (Tack *et al*. 2014a,b)], *Ampelomyces* spread among conidia is likely to represent a major fitness cost to the mildew. Thus, *Ampelomyces* infection represents a threat to the mildew's fitness by limiting both within‐season spread and between‐season survival.

*Ampelomyces* strains originating from different host mildew species have been noted to vary in their infection severity (Angeli *et al*. 2012b; Legler *et al*. 2016). However, phenotypic variation among *Ampelomyces* isolates from the same host mildew at local spatial scales (e.g. within metapopulations) has not been explored in a natural pathosystem. Nor have Pathogen^G^ × Hyperparasite^G^ interactions been shown to determine the outcome of infection. Previous work has found genetic variation among *Ampelomyces* isolates infecting multiple mildew host species (Kiss 1997; Liang *et al*. 2007). This variation has been linked with a broad host association using conserved rDNA ITS markers in some studies (Park *et al*. 2010), but multiple *Ampelomyces* genotypes have been found within single mildew species by others (Pintye *et al*. 2012). Moreover, microsatellite markers have revealed some divergence among *Ampelomyces* strains that infect phenologically distinct hosts (Kiss *et al*. 2011; Pintye *et al*. 2015). Ultimately, the degree to which *Ampelomyces* spp co‐evolve with specific mildew species and strains remains an open question. As *Ampelomyces* is dependent upon its mildew host for survival, it can complete its life cycle (infection to sporulation) within a single clonal cycle of its mildew host. However, the existence or timing of sexual reproduction by *Ampelomyces* is currently unknown, and thus, the true relative generation times of both organisms are difficult to calculate.

### Fungal and plant material

*Pl. lanceolata* clones were kept in greenhouse conditions at 20 ± 2 °C in 1:1 mixture of potting soil and sand. In the experiments described below, a single clonal plant genotype was used to control for any plant–host genotypic effects on the pathogen–hyperparasite interaction. This plant genotype had been in culture since 2012 and had shown a broad susceptibility to multiple pathogen isolates. It is also allopatric to all fungal isolates used in these experiments.

*Po. plantaginis* strains were collected from Åland in 2013 and 2015 (see Table S1, Supporting information) as infected leaf lesions. Isolates went through at least three rounds of purification with single‐colony transfers following isolation from the field and were subsequently maintained on detached *Plantago* leaves in a growth chamber at 20 ± 2 °C with 16:8 L:D photoperiod. Mildews were inoculated onto new maintenance leaves every 14 days.

*Ampelomyces* isolates used here were collected from *Po. plantaginis* infected leaves in Åland in 2014 and 2015 (see Table S1, Supporting information). The hyperparasite was maintained in pure culture on custom agar media (3 g NaNO_3,_ 1 g K_2_HPO_4_, 0.5 g KCl, 0.5 g MgSO_4_, 30 g C_12_H_22_O_11,_ 115 g Agar & Barley malt, litre^−1^) in darkness at ambient temperature. Isolates were turned over onto new media every 8 weeks.

### Laboratory tests for G×G interactions and local adaptation

We conducted two laboratory inoculation studies to determine whether hyperparasite infection outcome is determined by either organism's genotype and to investigate whether there is evidence for local adaptation between the organisms.

Both experiments were carried out on detached *Pl. lanceolata* leaves placed onto moist filter paper in a Ø 9‐cm petri dish. *Po. plantaginis* conidial spores were taken from 14‐day‐old *c*. 1 cm^2^ lesions and evenly spread across the recipient leaf surface with a fine, sterile paintbrush. Previous work on this system has demonstrated that this method produces repeatable infection outcomes (Laine 2004; Susi & Laine 2013). Growth of the pathogen was tracked every 2 days starting at 6 days post‐mildew inoculation (DPM) until 16 DPM. *Ampelomyces* isolates were inoculated onto mildew‐infected leaves at 8 DPM as a 70 ± 2 μL spray of *Ampelomyces* spore suspension in filter‐sterilized H_2_O (1 × 10^6^ spore/mL). Spore suspensions were obtained by scraping the surface of four 6‐week‐old *Ampelomyces* agar into filter‐sterilized H_2_O. Spore concentrations were assessed with a haemocytometer, and the suspensions were then diluted to the required concentration.

*Po. plantaginis* development was recorded on a scale modified from Bevan *et al*. (1993): 0: no growth, 1: spore germination and hyphae visible under a dissecting microscope, 1.5: mycelia with very few conidia visible only under a dissecting microscope, 2: mycelia visible to naked eye and sparse sporulation visible under a dissecting microscope, 3: abundant sporulation and lesion size <0.5 cm^2^, 4: abundant sporulation and lesion size >0.5 cm^2^. Established *Ampelomyces* infections produce distinctive brown spore structures within their host's conidia called pycnidia. Leaves were observed every 48 h with a dissecting microscope for the appearance of pycnidia starting at 10 DPM until the 16 DPM. *Ampelomyces* infection was scored with a modified version of the scale reported in Falk *et al*. (1995): 0: no pycnidia observed, 1: 1–20 pycnidia in each *Ampelomyces* cluster appearing, 2: 20–50 pycnidia in each powdery mildew lesion or between 30 and 50% of powdery mildew covered, 3: 50+ pycnidia in each powdery mildew lesion or >50% of powdery mildew covered. Levels 2 and 3 on this scale can reflect either a set number of *Ampelomyces* pycnidia or an estimate of pycnidia coverage of the mildew lesion. In this way, the scale controls for the different amounts of powdery mildew tissue available for the hyperparasite to infect; that is, small mildew lesions can still support a level‐3 hyperparasite infection even if there is not enough tissue to produce 50 +  pycnidia. *Ampelomyces*‐negative control leaves infected with just *Po. plantaginis* were sprayed with H_2_O only. Negative controls were observed to confirm the absence of *Ampelomyces* contamination and to follow the growth of the powdery mildew without *Ampelomyces* infection. Both experiments were run in two blocks, 24 h apart.

##### Experiment 1: Allopatric variation

In the first experiment, we investigate how much *Ampelomyces* and *Po. plantaginis* genotypes, and their potential interaction, determine hyperparasite infectivity and infection severity. We used a fully reciprocal cross‐inoculation design to challenge five distinct genotypes of *Po. plantagins* that were collected in 2013 with three *Ampelomyces* isolates collected in 2014. All five mildews and three *Ampelomyces* were collected from different populations, and all pathogen–hyperparasite combinations were also allopatric. Each combination was replicated 36 times at the outset of the experiment, although this was eroded in some treatments if host leaves died or the mildew became contaminated during the course of the experiment (see Table S2, Supporting information). The spatial origins of the strains used are given in Table S1 (Supporting information). Mildew genotypes [typed at 19 SNP loci detailed in Table S3 (Supporting information) (Tollenaere *et al*. 2012)] are given in Table S4 (Supporting information).

##### Experiment 2: Local adaptation

The second inoculation experiment was designed to test for local adaptation between mildew and *Ampelomyces*. We cross‐inoculated six *Ampelomyces* isolates onto 12 powdery mildew strains, representing seven mildew populations (see Table S1, Supporting information). All *Ampelomyces* isolates were exposed to at least one sympatric mildew isolate (from the same population) and all allopatric mildews. All *Po. plantaginis* and *Ampelomyces* strains used in this experiment were collected in September 2015, and so are temporally contemporary. Each mildew–hyperparasite isolate combination was replicated between five and 24 times once any contaminated or failed mildew‐inoculations were trimmed from the data (see Table S2, Supporting information).

### Assessing metapopulation‐wide and among‐population Ampelomyces prevalence

To assess patterns of hyperparasite prevalence at variable spatial scales, we conducted two sampling regimes in the Åland archipelago in the summer and autumn of 2014.

**Figure 1:**
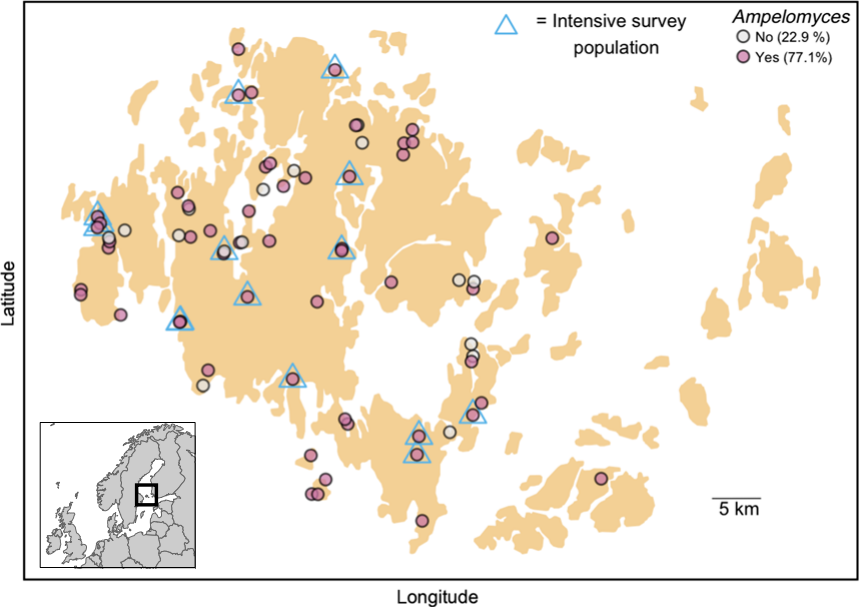
*Ampelomyces* is common at the metapopulation level. Map of the 70 *Po. plantaginis* populations in Åland that were screened during 2014. Of these, 54 were found to be infected by the *Ampelomyces* hyperparasite (pink circles). The 15 sites indicated with blue triangles were intensively surveyed across the whole summer of 2014.

In September 2014, 70 *Po. plantaginis* populations were surveyed and screened for *Ampelomyces* as part of an ongoing large‐scale metapopulation survey (Ojanen *et al*. 2013; Jousimo *et al*. 2014) (Fig. 1). First, survey assistants scored each pathogen population for host plant abundance and mildew coverage. Following positive identification of mildew, populations were revisited a few days later and up to five leaves from five infected plants at ≥1 m apart were collected, dried and processed for DNA extraction. Prior to drying, up to ten leaves from each population were examined under a dissecting microscope for the presence of *Ampelomyces* pycnidia. When found, these were isolated onto cell‐free agar culture for experimentation (see Fungal and Plant material section above). All leaf samples were screened for *Ampelomyces* infection using the qPCR method described below, and any mildew population exhibiting at least one *Ampelomyces*‐positive sample was considered to be an infected population.

### Spatial and host genetic factors as determinates of within‐population hyperparasite spread

To assess within‐population hyperparasite prevalence patterns, we intensively sampled fifteen discrete populations of *Po. plantagins* infecting *Pl. lanceolata* up to eight times from early July to late August 2014. These populations were selected based upon their previous mildew infection status; that is, they have been infected in the previous year, and their location within the Åland archipelago; they covered a broad range of geographical regions (Fig. 1, blue triangles).

At each survey time, a maximum of thirty mildew‐infected *Plantago* plants, that were at least 1.5 m apart, were identified and scored for the presence/absence of mildew. We also scored the coverage of infected and uninfected *Plantago* in a 3 m diameter around the focal plant. Plant positions were recorded using GPS triangulation that was then corrected by eye on detailed topographical maps in the field‐site. Up to five infected leaves were sampled from each plant throughout the survey period. We scaled the number and timing of samples depending upon the mildew infection severity of the plant, to avoid removing the mildew from the field completely. Leaf samples were dried prior to DNA extraction and subsequent SNP genotyping of the mildew and qPCR screening for *Ampelomyces* (see below for molecular methods).

From these survey data, we calculated indices of within‐population connectivity (*S*_*i*_) to mildew‐infected sites and *Ampelomyces*‐infected sites for each focal plant at each survey time point. First, we calculate connectivity to all mildew‐infected plants, regardless of hyperparasite infection status, as a measure of pathogen aggregation and dispersal potential within the population. This is given by

$$S_{i}=\sum p_{j}\text{exp}\;\left(-\alpha d_{ij}\right),$$

where *d*_*ij*_ is the Euclidian distance between focal plants *i* and *j*. We estimate dispersal distance (1/α) as 2 m, based on model simulations (Ovaskainen & Laine 2006) and empirical demonstrations (Tack *et al*. 2014a) that show the majority of *Po. plantaginis* spores land within 1 m of a progenitor infection and that dispersal over 2 m is extremely rare. *p*_*j*_ is the abundance of powdery mildew infection at location *j* (percentage coverage of *Pl. lanceolata* within a 1.5 m radius of the focal plant × the percentage of those *Pl. lanceolata* infected with powdery mildew). Our second index describes the connectivity of focal plants to *Ampelomyces*‐infected sites within the population by including the binary response of *Ampelomyces* (1 = infected, 0 = uninfected) as a multiplier of *p*_*j*_. We assume that *Ampelomyces* has at least a similar dispersal kernel to its host, and that the extent of mildew coverage also represents the maximum possible *Ampelomyces* coverage in a given focal circle, and thus, we keep α and *p*_*j*_ constant in both calculations. The square root of both indices is used in analyses.

To reduce the dimensionality of our data, we chose to estimate *Ampelomyces* prevalence at the ‘peak epidemic’ survey time for each focal plant, and thus, we took the maximum values found for all the variables mentioned above for each focal plant in every population. In this way, we account for the epidemics in each of the 15 populations occurring at different rates. Furthermore, given that *Ampelomyces* is dependent upon its host mildew for spread and survival, peak epidemic phase more realistically represents the risk of *Ampelomyces* invasion than, for example, the mean values for these metrics or by choosing an arbitrary time point at which to analyse all the data.

### Genotyping of powdery mildew hosts

DNA from leaf samples was extracted using E.Z.N.A plant DNA extraction kit at the institute of biotechnology, University of Helsinki. Samples were subsequently genotyped at 19 SNP loci (see Table S3, Supporting information) with the Sequenome iPlex platform at the Finnish Institute of Molecular Medicine Finland (FIMM) (see Tollenaere *et al*. 2012 for more details). SNP calling was performed with typer4 software (Table S3, Supporting information). Because we sampled the haploid phase of *Po. plantaginis*, samples which were called as heterozygous for any locus are determined to be coinfected with at least two mildew multilocus genotypes. Any sample that failed to call all 19 SNP loci was removed from genotype analysis.

To analyse *Ampelomyces* presence by MLG diversity, we used the ‘poppr’ package in r (cran core team) to correct for the clonal nature of the mildew and to calculate indices of MLG diversity and richness at the population level. Several indices of MLG diversity were calculated for each of these 15 populations, all of which showed strong colinearity. For this reason in our analyses, we chose to use only Shannon's H index of diversity and estimated MLG richness based on rarefaction curves (*N *=* *10) as computed by the ‘poppr’ package.

### Screening for *Ampelomyces* infection

Because only heavily established *Ampelomyces* infections can be observed by eye, and microscope detection is extremely laborious, we used an *Ampelomyces* ‐specific qPCR screening technique developed by (Tollenaere *et al*. 2014) to detect hyperparasite infections in the field. Briefly, each sample was run for 30 cycles (30 s at 95 °C, 30 s at 60 °C and 30 s at 72 °C) with the primers AmpITS_F (GCTGCCAATTGCTTTGAGAT) and AmpITS_R (GATGAAGAACGCAGCGAAAT) which target an *Ampelomyces‐*specific sequence within the rDNA ITS gene were used. This was followed by a melting curve analysis from 45 °C to 95 °C by 0.5 °C increment every 5 s. Samples with a mean *C*_T_ value ≤24 (over three replicate reactions with SD < 0.2) were determined to possess an established *Ampelomyces* infection (see Supporting information of Tollenaere *et al*. 2014 for establishment of this threshold).

### Statistical analyses

Statistical analyses were conducted in r (R core team 2016), and used additional packages ‘lme4’, ‘ordinal’, ‘poppr’, ‘mgcv’ and ‘car’. We broadly analysed our data within the generalized linear, generalized additive and cumulative link model frameworks. Minimum adequate models were derived from saturated models through stepwise simplification and selection based on likelihood ratio or χ^2^ tests of nested models (Crawley 2012). Nonsignificant factors are reported as the output of these model comparisons. The effect of significant independent variables is derived from analysis of the minimum adequate model with the ‘car’ package where possible or through model simplification when not. Where necessary, overdispersion was tested and accounted for by fitting a cloglog link function (fixed‐effect models) or an observation‐level random effect (mixed‐effect models). Before analysing our laboratory experiment, the data were trimmed to remove any leaf on which powdery mildew had not sporulated by 12 DPM, as these were regarded as failed pathogen inoculations which were unable to host *Ampelomyces* infection.

*Ampelomcyes* infectivity (pycnidia present at 16 DPM) in both inoculation experiments was modelled with mixed‐effect GLMs with a binomial distribution of errors and logit link function using package ‘lme4’. Mildew identity, *Ampelomyces* identity, their interaction and mildew growth at the time of hyperparasite inoculation (8 DPM) were coded as fixed effects. Experimental block was included as a random intercept. To test for local adaptation in our second experiment, we included a ‘sympatry/allopatry’ fixed effect to our model and furthermore included ‘sympatry/allopatry’ nested within ‘*Ampelomyces* strain identity’ as an additional random effect to account for overall variation in infectivity among *Ampelomyces* strains. Models of our second experiment failed to converge due to rank deficiencies if we included the interaction between mildew and *Ampelomyces* strains. Therefore, we included a separate factorial variable with a level for each pathogen–hyperparasite combination as a fixed effect to account for variation among strain combinations.

*Ampelomyces* infection severity was analysed for all lesions on which *Ampelomyces* infection was observed. These data were analysed with a cumulative link mixed‐effect model (clmm) to account for the ordinal nature of the *Ampelomyces* infection severity scale. Similar model structures as before were used but with *Ampelomyces* severity level (1–3) as the dependent variable and replacing mildew growth at 8DPM with mildew growth at the time of scoring (16DPM).

To analyse the variation in among‐population *Ampelomyces* prevalence in the field, we use a generalized linear model with a quasibinomial distribution of errors to account for overdispersion of the response variable. The proportion of leaf samples hosting *Ampelomyces* in each population was set as the dependent variable. Fixed independent variables were: estimated mildew MLG richness, Shannon's H index of MLG diversity, the proportion of leaf samples presenting signals of coinfection (heterozygous SNP calls at ≥1 loci), mean mildew coverage across focal plants in the population and the population's connectivity index relative to the entire pathogen metapopulation (as calculated in Penczykowski *et al*. 2014).

To assess patterns of within‐population prevalence of *Ampelomyces,* we analysed presence/absence of the hyperparasite in each leaf sample within a generalized additive model framework. We include two measures of hyperparasite spatial clustering in our analyses. First, within‐population connectivity of *Ampelomyces*‐infected plants is included as a fixed effect to test whether the hyperparasite is aggregated within its already aggregated mildew host. That is to say, does the *Ampelomyces* infection prevalence across neighbouring plants influence the probability that it will infect a given focal plant? Second, we include the spline of each focal plant's longitude and latitude as an additive fixed effect to account for spatial variation in infection risk across each population. That is to say, are *Ampelomyces* infections aggregated in space irrespective of how many hyperparasite infected and uninfected plants are in that location? We also include the maximum‐recorded amount of mildew surrounding each focal plant to determine whether relative pathogen abundance determines *Ampelomyces* infection success. We include focal plant identity nested within‐population identity as a random intercept to account for repeated sampling of the same host plants and the aggregation of host plants within discrete populations in the archipelago. Because our field data contain a large number of rare and singleton MLG types (75 MLGs, see Fig. 5D), we were unable to incorporate this information as a multi‐level factor in the analysis detailed above. Instead, we performed a second analysis on a truncated data set from which any sample with a rare MLG (<5 instances found in the full dataset) or evidence of coinfection was removed. This resulted in a reduced data set representing samples from 20 MLG types. Otherwise, the initial model structure for this analysis was the same as described previously. Numerical covariates in both of these analyses were centred and scaled before they were modelled.

## Results

### Hyperparasite and pathogen genotype combination determines infection outcome

**Figure 2:**
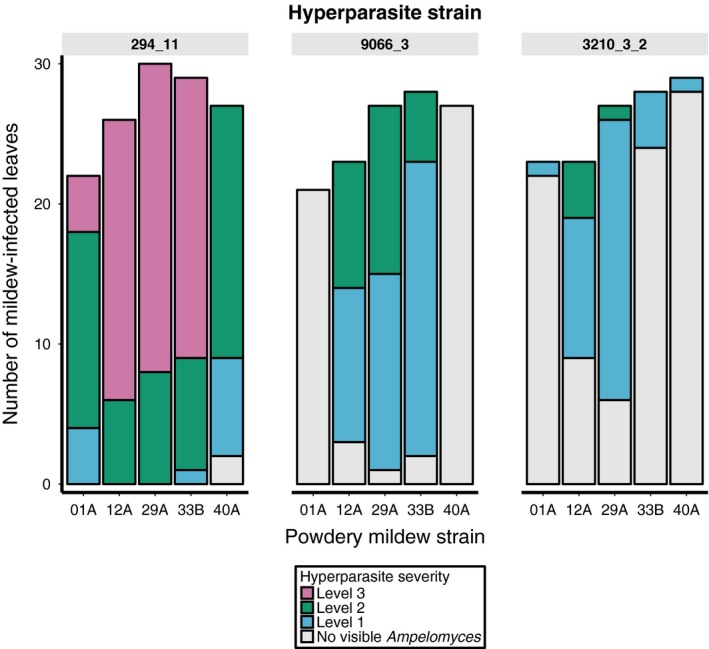
Hyperparasite infectivity and infection severity varied among pathogen–hyperparasite genotype combinations. Hyperparasite strain 294_11 performed better than both other strains in terms of both the proportion of mildew lesions it infected, and the extent of infection after 16 days of growth. Mildew strains also vary in their susceptibility to hyperparasite attack, which is dependent upon the identity of the hyperparasite. For instance, mildew 33B is highly susceptible to 294_11 and 9066_3, but is able to resist 3210_3_2.

Our laboratory cross‐inoculation experiment of allopatric pathogen–hyperparasite isolates revealed that *Ampelomyces* infectivity was significantly dependent upon the mildew genotype (χ^2^ = 38.13, d.f. = 5, *P *<* *0.001), hyperparasite genotype (χ^2^ = 51.57, d.f. = 3, *P *<* *0.001) and their interaction (χ^2^ = 19.21, d.f. = 8, *P *=* *0.0138) (Fig. 2). Hyperparasite infectivity was also significantly, positively correlated with the developmental stage of the mildew lesion at the time of hyperparasite inoculation (χ^2^ = 9.113, d.f. = 1, *P *=* *0.003). The severity of *Ampelomyces* infection was analysed at 16 DPM, at which point there was a significant effect of both mildew and hyperparasite strain identity (mildew genotype: LRT = 94.289, d.f. = 4, *P *<* *0.001, hyperparasite genotype: LRT= 257.2, d.f. = 2, *P* < 0.001), but no significant interaction between these factors (LRT=1.77, d.f. = 2, *P* = 0.2). *Ampelomyces* infection severity was also positively correlated with the mildew infection severity at the time of scoring (LRT = 27.714, d.f. = 1, *P *<* *0.001).

### Hyperparasite vary in their degree of local adaptation to their mildew host

**Figure 3:**
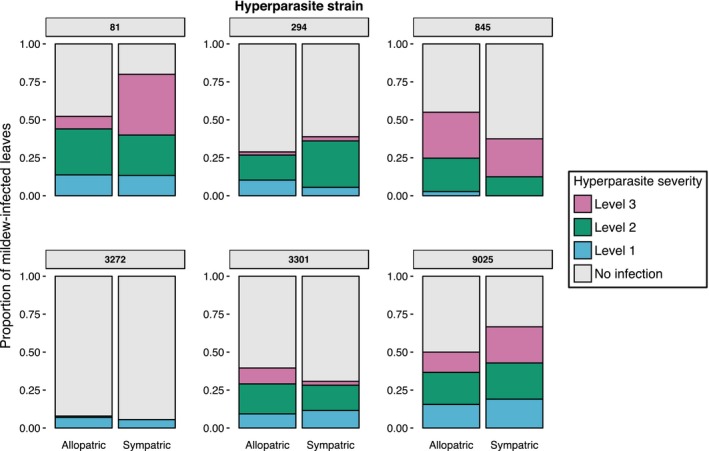
*Ampelomyces* can be locally adapted to its mildew host. Hyperparasite infectivity and infection severity strongly varied among hyperparasite strains (individual plots) and between sympatric/allopatric mildew hosts. Statistical analysis suggests that *Ampelomyces* was more infective against sympatric mildew than the mean of all allopatric combinations; however, the presence and intensity of this effect varied greatly among hyperparasite genotypes. This could indicate that selection for local adaptation varies in strength across the metapopulation, or that the strength of selection is highly dynamic over evolutionary time.

Our test for local adaptation found that hyperparasites were more successful at infecting sympatric pathogen strains than allopatric ones (χ^2^ = 4.69, d.f. = 1, *P *=* *0.03) (Fig. 3). However, there was variation in this trend, with a significant impact of mildew genotype (χ^2^ = 55.609, d.f. = 6 *P *<* *0.001) and *Ampelomyces* strain (χ^2^ = 23.74, d.f. = 5 *P *<* *0.001) on hyperparasite infectivity. There was no significant effect of mildew and *Ampelomyces* strain combination on hyperparasite infectivity (χ^2^ = 3.3, d.f. = 5, *P* = 0.654). As above, the lesion size of the mildew at the time of *Ampelomyces* inoculation had a significant, positive impact on hyperparasite infection success (χ^2^ = 33.06, d.f. = 1, *P *<* *0.001). Hyperparasites significantly affected sympatric mildew isolates more severely than allopatric ones (LRT = 6.547, d.f. = 1, *P *=* *0.011), and infection severity was also significantly dependent upon the identity of both the mildew (LRT = 25.23, d.f. = 5, *P *<* *0.001) and the *Ampelomyces* (LRT = 19.67, d.f. = 6, *P* = 0.003).

### Hyperparasite prevalence varies at different spatial scales

Metapopulation‐scale sampling of the Åland archipelago found that 54 of 70 (77.1%) *Po plantaginis* populations supported *Ampelomyces* infection, indicating that the hyperparasite is widespread across the archipelago. However, in the 15 populations that were intensively sampled throughout the epidemic season, we found that hyperparasite prevalence ranged between 0 and 45%. *Ampelomyces* prevalence in these populations was not significantly associated with mildew genetic diversity (χ^2^ = −0.006, d.f. = 1, *P* = 0.9), MLG richness: (χ^2^ = −0.052, d.f. = 1, *P* = 0.91) or mildew population size (χ^2^ = −0.436, d.f. = 1, *P* = 0.729). However, the proportion of leaf samples supporting coinfecting mildew MLGs and the connectivity of the populations within the mildew metapopulation have a marginally positive effect on hyperparasite prevalence (mildew coinfection: χ^2^ = −11.26, d.f. = 1, *P* = 0.067, population connectivity: χ^2^ = −12.232, d.f. = 1, *P* = 0.056).

### Spatial structure determines within‐population prevalence

**Figure 4:**
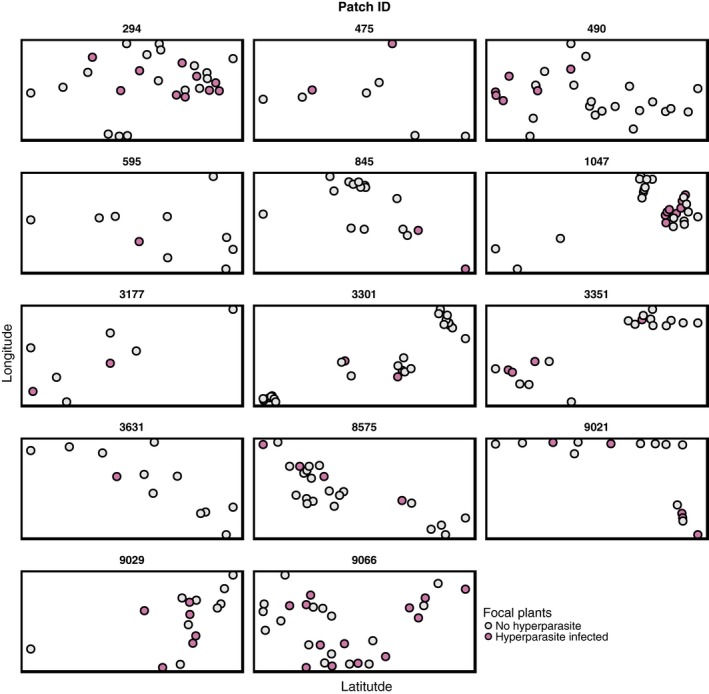
*Ampelomyces* infections are spatially clustered among mildew‐infected plants within populations. Facets represent geometric area of the 14 intensively surveyed *Po. plantaginis* populations in which at least one mildew‐infected leaf supported a hyperparasite infection. Circles denote the locations of mildew‐infected *Plantago lanceolata*. Pink circles denote host plants from which at least one mildew sample was found to support the hyperparasite.

**Figure 5:**
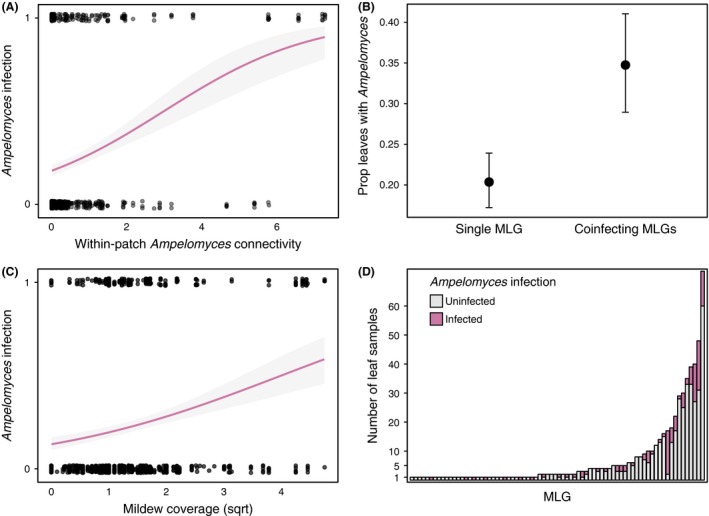
Factors influencing within‐population *Ampelomyces* prevalence. *Ampelomyces* infections are significantly positively correlated with the connectivity of *Ampelomyces*‐infected plants (A) and also significantly more common on leaves supporting >1 mildew MLG (B). There is a positive, but not significant correlation between the amount of mildew surrounding a focal plant and its probability of supporting *Ampelomyces* (C). Mildew MLG identity does not significantly predict *Ampelomyces* presence, although hyperparasite infectivity does vary across MLGs (D).

Analysis of our intensive field surveys (Fig. 4 and Table 1) data revealed that a focal plant's connectivity to *Ampelomyces*‐infected plants within a population positively correlated with the probability that it also hosts the hyperparasite (z = 5.310_731_ *P *<* *0.001, Fig. 5A). In addition, leaves that supported multiple co‐infecting mildew MLGs were significantly more likely to also host the hyperparasite (z = 2.104_731_,_1_, *P* = 0.032, Fig. 5B). Furthermore, the spatial location of plants within populations significantly affected their probability of hosting *Ampelomyces* (approx. significance of smooth terms: χ^2^ = 8.101, e d.f. = 2, *P *=* *0.02, Fig. 4). However, patterns of within‐population *Ampelomyces* occurrence were not significantly associated with the amount of mildew surrounding focal plants (χ^2^ = 1.1027, d.f. = 1, *P *=* *0.293, Fig. 5C). In addition, within‐population connectivity of mildew‐infected plants also has no significant bearing on whether they were infected with the hyperparasite (χ^2^ = 3.176, d.f. = 1, *P* = 0.075).

**Table 1:** Factors contributing to *Ampelomyces* infection in natural field populations analyzed with generalised linear additive models

	Estimate ± SE	Test statistic	*P*‐value
Variables dropped through model selection			
Focal circle mildew coverage_1,707_	—	χ^2^ = 1.107	0.293
Mildew connectivity_1,707_	—	χ^2^ = 1.722	0.189
Variables retained after model selection			
Intercept_1,733_	−2.329 ± 0.231	*z *=* *−10.075	**<0.001**
Coinfection_1,733_	0.649 ± 0.303	*z *=* *2.141	**0.032**
*Ampelomyces* connectivity_1,733_	1.273 ± 0.215	*z = *5.931	**<0.001**
*Spline*(long, lat)		χ^2^ = 8.101	**0.017**
Random effects	Variance		
Focal plant (within population)_259,707_	4.6	—	—
Population_14,707_	0.025	—	—

Significant *P*‐values (<0.05) are in bold.

Separate analysis of *Ampelomyces* infection in the most common MLG types in our data set revealed no significant association between mildew identity and hyperparasite infection (χ^2^ = 9.46, d.f. = 1, *P *=* *0.221). However, we still see a significant effect of within‐population *Ampelomyces* connectivity (z = 5.222_352_, *P *<* *0.002) even in this truncated data set.

## Discussion

Understanding how pathogens and parasites interact with their own natural enemies will be essential if we are to effectively predict and control outbreaks of infectious disease. Here, we assess the contribution of both genetic and spatial components to a hyperparasite–pathogen interaction. We show that under laboratory conditions, the establishment and severity of a hyperparasite infection varies depending upon the identity of both antagonists, suggesting that main genotype and G×G dynamics govern the outcome of hyperparasitism. Furthermore, we find evidence for local adaptation of the hyperparasite to its sympatric pathogen host strain across a spatially fragmented landscape, although the strength of this varies across populations. However, when exploring hyperparasite–pathogen interactions in the field, we do not find a strict association of *Ampelomyces* with specific host strains. This result reinforces previous population genetic work which found that *Ampelomyces* strains are not strictly associated with their host lineage (Pintye *et al*. 2012). Rather, we find that hyperparasite infection is more likely when host genetic diversity is high, that is multiple mildew genotypes coinfect the same plant. Furthermore, we show that the hyperparasite can be spatially aggregated and relatively rare (<40% prevalence) within local populations of its pathogen host, but is very common at the metapopulation scale (77.1% of populations).

Our data suggest that *Ampelomyces* may be dispersal‐limited within pathogen populations, or sensitive to local environmental conditions. In contrast, *Ampelomyces* is relatively common at the metapopulation level. This may be partially explained by the timing of the respective surveys; July–August for within‐population prevalence, early September for the metapopulation‐wide survey. Although, an alternative explanation for this dichotomy is that *Ampelomyces* may be present upon multiple mildew hosts in Åland, which allows it to cover a large geographical area and to infect many *Po. plantaginis* populations despite having limited spread at the local level. Several studies have shown that many *Ampelomyces* isolates can readily host‐shift (Liang *et al*. 2007; Kiss *et al*. 2011; Angeli *et al*. 2012b), so the hyperparasite infections that we observe may be the result of a spillover from *Ampelomyces* reservoirs in other mildew species (Power & Mitchell 2004). However, this would not explain why we see limitations to *Ampelomyces* spread once it has spilled‐over to a *Po. plantaginis* population, nor would it explain why we see the same *Ampelomyces* isolate performing differently against different mildew hosts in the laboratory.

Interestingly, our laboratory and field observations present contrasting results regarding the importance of genetic factors in governing hyperparasite infection. Our laboratory experiments strongly suggest that both main genotype and G×G interactions dictate hyperparasite infectivity and infection severity. However, in the field we find no significant effect of mildew host genotype on *Ampelomyces* prevalence patterns. Rather, we find that the hyperparasite is spatially clustered among mildew‐infected plants, which alludes towards an environmentally derived barrier to infection spread. This discrepancy may indicate that *Ampelomyces* epidemiology in nature is strongly influenced by ecological factors before any genetic filtering can occur. Indeed, the degree of aggregation appears to vary among populations (Fig. 4), and so is likely to be a function of the multiple environmental factors that affect dispersal, such as prevailing wind strength and direction, rainfall, humidity and the presence of physical barriers. It is also possible that the severity and specificity of any G×G interactions and local adaptation effects are sensitive to such environmental factors. Similar issues have been encountered in other parasitic interactions, where the environment can influence the outcome of genetic interactions between organisms (Laine 2008; Bryner & Rigling 2011; Tack *et al*. 2015). Furthermore, the discrepancy between our laboratory and field observations may also be partially due to the sheer genetic diversity present in our pathogen populations. For instance, it is possible that several distinct mildew genotypes within a given population may be equally susceptible or resistant to the resident *Ampelomyces*. This could mask any signal of host association, particularly if many mildew genotypes are rare and thus underrepresented in our data.

Although we have found no evidence that the hyperparasite is associated with particular pathogen genotypes in nature, our field observations do demonstrate that *Ampelomyces* is most likely to be found on plants that are coinfected by multiple mildew genotypes. In addition, we found that the amount of mildew coinfection within a population positively correlated with the prevalence of the hyperparasite. Interestingly, this effect appears to be independent of the absolute amount of mildew available for the hyperparasite to infect, as there was no significant correlation between hyperparasite infection and pathogen coverage of either the focal plant or within a 3 metre radius. These results confirm a similar pattern found by (Tollenaere *et al*. 2014) and strongly suggest that some property of coinfecting pathogens makes them more suitable as hosts for the hyperparasite. If *Ampelomyces* infection is indeed determined in a G×G manner, as our experimental results suggest, then coinfections may simply double the probability that one of the two mildews present is a suitable host. Alternatively, mildew physiology may alter under coinfection in a way that increases their susceptibility to hyperparasite infection. For example, mildews may invest in sexual reproduction or competition with coinfecting conspecifics rather than defence against the hyperparasite.

Despite the lack of genetic filtering in our field observations, our laboratory results are, to our knowledge, the first to demonstrate any degree of genotype‐by‐genotype interaction and local adaptation in a horizontally transmitted hyperparasite system. G×G interactions and local adaptation are key processes driving disease dynamics and governing infection risks in natural pathogen populations (Lambrechts *et al*. 2006; Tack *et al*. 2012), but these processes require a degree of fidelity between host–pathogen strains to be maintained over evolutionary time. Many hyperparasites are likely to be under selection to utilize multiple host species and/or to disperse over long distances in order to maintain an effective population size (Nair *et al*. 2016). Thus, we do not necessarily expect G×G interactions or local adaptation to arise in hyperparasite systems. *Ampelomyces* has been shown to readily host‐shift among mildew species under laboratory conditions (Liang *et al*. 2007; Kiss *et al*. 2011; Angeli *et al*. 2012b), and molecular work indicates that strains are only very loosely associated with distinct mildew species (Liang *et al*. 2007; Park *et al*. 2010; Kiss *et al*. 2011). Therefore, our finding that *Ampelomyces* exhibits a G×G relationship within genetically distinct isolates of one species of powdery mildew is unexpected. Indeed, the only other evidence for a pathogen–hyperparasite G×G interaction has been found between a pathogenic fungus and a virus that can vertically transmit within lineages of its host, thus sidestepping the issue of host scarcity (Bryner & Rigling 2011). This may be testament to how rapidly co‐evolutionary forces can shape host–pathogen interactions and generate diversity within populations, even at higher trophic levels.

Our laboratory experiments also revealed a signal of local adaptation of the hyperparasite to its sympatric mildew host when compared to the average of allopatric combinations. This effect was seen both in measures of infectivity and infection severity, but was highly variable among populations. Similarly to our laboratory evidence for G×G dynamics, the extent to which *Ampelomyces* local adaptation governs hyperparasite epidemiology in nature is unclear and may be minimal. However, this does allude to a co‐evolutionary relationship between pathogen and hyperparasite. Co‐evolutionary relationships such as these can be highly dynamic over short periods of time and across metapopulations. Thus, the variation in hyperparasite adaptation that we see among populations may be a direct result of temporal fluctuations in the strength and synchrony in co‐evolutionary selection (Thompson 2005; Blanquart *et al*. 2013; Koskella 2014). Combining both a sympatry/allopatry and time‐shift approach to future studies in this system may reveal the evolutionary dynamics more fully.

Several studies have documented that the infection severity of *Ampelomyces* isolates can vary depending on the species of mildew host (Liang *et al*. 2007; Kiss *et al*. 2011; Angeli *et al*. 2012b), but our study is the first to robustly demonstrate phenotypic variation of the hyperparasite within a single host species at an intermediate spatial scale (5–50 km). Previous work on this system has found extremely low levels of diversity in the *Ampelomyces* strains infecting *Po. plantaginis* using conservative genetic markers (Tollenaere *et al*. 2014). In the light of this, our findings suggest that relatively rapid co‐evolutionary processes are able to maintain phenotypic diversity within highly related groups of hyperparasite strains. Therefore, to fully explore within‐*Ampelomyces* variation, and thus accurately track mildew–hyperparasite associations across the natural metapopulation, future studies should utilize population genetic techniques that can detect variation in rapidly evolving loci. A suite of *Ampelomyces* genes recently identified in host recognition and infection present a promising resource for such work (Siozios *et al*. 2015).

Our results also demonstrate that powdery mildew strains of a single species deriving from a single metapopulation vary in their susceptibility to the hyperparasite. Whether this variation represents adaptive resistance to *Ampelomyces* attack, or is a by‐product of the variation present in the *Po. plantaginis* metapopulation is unknown. Both empirical and theoretical work have shown that variation in susceptibility to natural enemies across spatially structured populations can fundamentally alter epidemiological and evolutionary patterns of infectious disease (Salvaudon *et al*. 2008; Laine 2011; Jousimo *et al*. 2014). However, this has not been explicitly considered in the case of pathogens that must both defend against hyperparasite attack and also infect and spread among their own hosts. The consequences of evolving resistance to hyperparasites for pathogen epidemiology, and even the existence of nonadaptive variation in susceptibility among pathogen populations, are of significant interest when considering hyperparasites as effective biocontrol measures (Parratt & Laine 2016).

The risk of disease incidence and severity of infectious outbreaks can vary greatly over spatially structured host populations (Ostfeld *et al*. 2005). Determining the factors, both genetic and ecological, that drive this variation is imperative if we are to successfully predict and tackle the emergence of dangerous human, agricultural and wildlife pathogens. Given that hyperparasites can fundamentally alter disease virulence (Abo‐Foul *et al*. 1996; Nuss 2005), epidemiology (Verhaar *et al*. 1996; Andersen *et al*. 2012; Springer *et al*. 2013; Tollenaere *et al*. 2014) and evolution (Taylor *et al*. 1998), they may represent a key top‐down moderator of disease dynamics. Here, we have shown that hyperparasite infectivity and virulence can vary greatly across intermediate spatial scales due to genetic variation in both the hyperparasite itself and its host. We also show that pathogen spatial configuration is still a key determinant of local‐level hyperparasite spread in nature, even if hyperparasites are ubiquitous in the environment. These findings have implications for selecting hyperparasite strains when considering the use of these organisms as biocontrol measures for human and agricultural epidemics (Swinton & Gilligan 1999; Nobrega *et al*. 2015).

The study was conceived and designed by S.R.P. and A.‐L.L. Intensive field surveys were designed and conducted by S.R.P., R.M.P. and B.B. Pathogen SNP genotyping and analysis was conducted by S.R.P. and B.B. Laboratory experiments and data analyses were conducted by S.R.P. and R.M.P. The main manuscript was written by S.R.P. and A.‐L.L., and all authors read and contributed to the final draft.

## Data accessibility

All data presented in this manuscript are available on the Dryad digital repository: doi:10.5061/dryad.5fh23.

## Supporting information


**Table S1.** Fungal strains used in laboratory experiments.

**Table S2.** Replication within each treatment of the two inoculation experiments.

**Table S3.** SNP markers and their typer4 calling parameters used to genotype Podosphaera plantaginis.

**Table S4.** MLG genotypes of *Podosphaera plantaginis* strains used in laboratory inoculation experiments.
